# Retraction: Chinese Herbal Medicine Suppresses Invasion-Promoting Capacity of Cancer-Associated Fibroblasts in Pancreatic Cancer

**DOI:** 10.1371/journal.pone.0313436

**Published:** 2024-11-01

**Authors:** The PLOS ONE Editors

After this article [1] was published, concerns were raised about results presented in Figs 2, 5, and S2. Specifically:

- In Fig 2 in [1], some panels appear similar to panels in Fig 3B in [2], and/or its Correction [3], specifically:
  ○ The Fig 2 #2 migration CAF (Ctrl-treated) panel in [1] appears similar to the Fig 3B Tca8113 Empty-Vector panel in [2] and [3].
  ○ The Fig 2 #2 invasion NF panel in [1] appears similar to the Fig 3B KB SB225002(0.5μm) panel in [2] and [3].
  ○ The Fig 2 #2 invasion CAF (Ctrl-treated) panel in [1] appears similar to the Fig 3B KB Empty-Vector panel in [2] and [3].
  ○ The Fig 2 #2 invasion CAF (QYHJ-treated) panel in [1] appears similar to the Fig 3B Tca8113 SB225002(0.5μm) panel in [3].
- In Fig 5 in [1]:
  ○ The left half of the anti-CXCR1 Ab (CXCL8) panel in the third set of invasion experiments (for which CXCL8 was added) appears similar to the right half of the vehicle panel in the same set of experiments. These areas also appear similar to the right-hand region of the Anti-CXCR1 Ab panel in Fig 4b in [4]. The right-hand region of the Anti-CXCR1 Ab (CXCL8) invasion panel appears similar to the Vehicle panel of Fig 4b of [4].
  ○ The migration panels for the third set of experiments (for which CXCL8 was added) appear similar to the migration panels in Fig 4a in [4], which represent the same experimental conditions.
  ○ The right half of the Anti-IgG Ab (CXCL8) panel in the third set of invasion experiments appears similar to the Anti-IgG Ab panel in Fig 4b in [4].
- In Fig S2, the bottom of the #1 CAF (Ctrl-treated) invasion panel appears similar to the top of the #2 CAF (Ctrl-treated) invasion panel.

In response to queries about these experiments, the corresponding author stated that the above-listed panels in Fig 5 of [1] are incorrect. They also stated that the above-listed panels in Fig S2 are incorrect. For Fig 2, they stated that all panels in Fig 2 are correct, but noted that different research groups at the same institution used the same microscope equipment and computers.

The corresponding author provided the underlying data for the charts in Fig 2, which was reviewed by an Academic Editor who noted that for the upper chart, the difference in results for NF versus Ctrl-treated CAF groups are not statistically significant and that significant differences were observed only for NF versus QYHJ-treated CAF and Ctrl-treated CAF versus QYHJ-treated CAF in this experiment. The corresponding author stated that there is an error in the upper panel of Fig 2, which incorrectly showed the difference for NF versus Ctrl-treated CAF groups as not statistically significant.

In light of the extent of the issues listed above that raise concerns about the reliability and validity of the reported results and conclusions, the *PLOS ONE* Editors retract this article.

PW did not respond to the final editorial decision. LC, CQ, HC, LX, QQ, JL, KW, ZM, ZC, and LL either did not respond directly or could not be reached.

## References

[1] Chen L, Qu C, Chen H, Xu L, Qi Q, Luo J, et al. (2014) Chinese Herbal Medicine Suppresses Invasion-Promoting Capacity of Cancer-Associated Fibroblasts in Pancreatic Cancer. PLoS ONE 9(4): e96177. 10.1371/journal.pone.0096177 24781445 PMC4004556

[2] J Oral Pathol Med (2014) 43: 658–666 24953191 10.1111/jop.12189

[3] (2022), Corrigendum. J Oral Pathol Med, 51: 217–217. 10.1111/jop.13272 35170811

[4] Chen L., Fan J., Chen H. et al. The IL-8/CXCR1 axis is associated with cancer stem cell-like properties and correlates with clinical prognosis in human pancreatic cancer cases. Sci Rep 4, 5911 (2014). 10.1038/srep05911 25081383 PMC4118151

